# Case reports of identical twins with developmental and epileptic encephalopathy with STXBP1 gene mutations for whom different CBD supplementations were markedly effective

**DOI:** 10.1016/j.ebr.2024.100720

**Published:** 2024-10-24

**Authors:** Yuji Masataka, Naoko Miki, Kozo Akino, Hitoshi Yamamoto, Ichiro Takumi

**Affiliations:** a Department of Neurology, Kumamoto Seijo Hospital, Kumamoto, Japan; b General Incorporated Association Green Zone Japan, Saitama, Japan; c General Incorporated Association Japan Clinical Association of Cannabinoids, Kanagawa, Japan; d Member of the House of Councillors (District of Fukuoka Prefecture), Tokyo, Japan; e Department of Pediatrics, St. Marianna University School of Medicine, Kanagawa, Japan; f Department of Neurosurgery, St.Marianna University School of Medicine, Kanagawa, Japan

**Keywords:** CBD, Cannabidiol, Epilepsy, Broad-spectrum, Cannabis

## Abstract

Cannabidiol (CBD) is a compound found specifically in the cannabis plant. Although a clinical trial for intractable epilepsy started in Japan in 2023, it is also available in the market as a dietary supplement. Herein, we report two cases of identical twins with developmental and epileptic encephalopathy with *STXBP1* gene mutation who achieved seizure suppression through different regimens of CBD supplementation. The observation that different trace ingredients produced different effects in patients with identical genetic backgrounds is a crucial finding that has implications for the future regulation and clinical application of cannabinoid products.

## Highlights

• Identical twins with intractable epilepsy achieved seizure suppression with CBD.
• CBD products that proved effective in each patient differed, despite their genetic similarity.
• Broad-spectrum CBD products may be effective in cases where CBD isolate products are ineffective.

## 1 Introduction

Cannabidiol (CBD) is a cannabinoid (CBs), a class of chemicals found specifically in cannabis plants, and its chemical structure was identified by Mechoulam et al. in Israel in 1963[1]. Unlike THC (Δ 9-tetrahydrocannabinol), the primary psychoactive constituent in the cannabis plant, CBD has neither psychoactive nor addictive effects and is widely distributed as a drug, food, and dietary supplement [2]. Epidiolex®, a CBD-based prescription drug, is approved in the U.S. and Europe for the treatment of Dravet syndrome, Lennox-Gastaut syndrome, and tuberous sclerosis[3].

The Japanese Cannabis Control Act excludes seeds and mature stalks of cannabis plants from the statutory definition of cannabis [4]. This is because when the law was enacted, mature cannabis stalks and seeds were cultivated in Japan as a source of fiber and food [5]. Consequently, CBD supplement products have been legally distributed since 2013, and these consumer products have been shown to suppress epileptic seizures [6].

Herein, we present two cases of identical twins with developmental and epileptic encephalopathy (DEE) with *STXBP1* gene mutation, who achieved seizure suppression through different regimens of CBD supplementation. Syntaxin-binding protein 1 (STXBP1; also known as MUNC18-1) is an essential component of the presynaptic neurotransmitter release mechanism. De novo heterozygous pathogenic variants of *STXBP1* are among the most prevalent causes of neurodevelopmental disorders, including intellectual disabilities and epilepsy [7].

### 1.1 Case 1

The patient was a 2 years and 9 months old female who majorly complained of convulsive seizures. The patient had an identical twin sister (Case 2) with a history of epilepsy. Born at 37 weeks and 3 days of gestation, she weighed 2748 g at birth and was the second child in a spontaneous monochorionic diamniotic twin pregnancy and spontaneous vaginal delivery. At 31 days of age, the patient began to experience spasms in her left upper limb. Following consultation with her primary care physician, she was admitted to University Hospital B for a thorough examination.

At the initial admission, the patient weighed 3434 g. She exhibited no external deformities and had normal muscle tone; however, clusters of spasms and tonic seizures lasting 20 seconds were observed. Blood counts and general biochemistry results were normal, and newborn screening revealed no congenital abnormalities. An MRI scan of the head showed no abnormal findings.

During this admission, phenobarbital (PB) was administered to her when the patient was 33 days old, which was followed by the addition of Valproic Acid (VPA), but the seizures persisted. Adrenocorticotropic hormone (ACTH) therapy was initiated when the patient was 54 days old. Despite discontinuing PB and increasing VPA dosage, along with the addition of Vitamin B6, Diazepam (DZP), clobazam (CLB), and topiramate (TPM), seizure suppression was not achieved, and the patient was transferred to a specialized facility, Hospital C.

**Figure 1:**
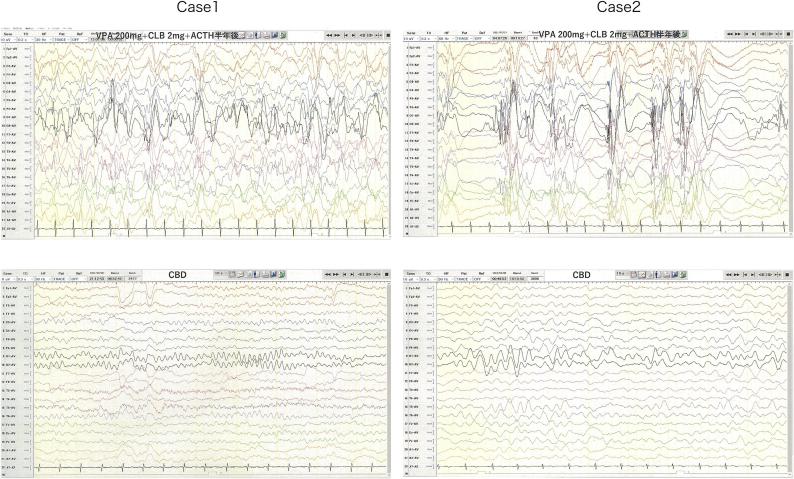
Electroencephalogram (EEG) before and after treatment Upper left: Case 1 (at 269 days old). Irregular bilateral wide-amplitude high-amplitude slow waves emerge, which are intermixed with non-rhythmic and asynchronous spiky waves. Lower left: Case 1 (at 770 days old). The EEG displays predominantly low to moderate amplitude theta waves. The occurrence of interictal epileptiform discharges has been significantly reduced. Upper right: Case 2 (at 269 days old). High-amplitude slow waves and spiky waves appear independently, without a discernible rhythm, and asynchronously. Lower right: Case 2 (at 770 days old). Following CBD treatment, the occurrence of interictal epileptiform discharges is markedly reduced.

At Hospital C, exome sequencing analysis identified a heterozygous STXBP1 variant, NM_003165: c.325+5G>A, in intron 5. VPA was changed from syrup to granules, and her seizures remained uncontrolled, even after discharge. Consequently, she was readmitted to University Hospital B. Long-term EEG, conducted when she was 269 days old, revealed hypsarrhythmia, and although a second ACTH treatment was recommended, her parents declined. Instead, they inquired about the use of CBD. Fig. 1

When the patient was 280 days old, her parents started giving her a CBD supplement 1 (dr. hennep Co. Ltd.) at a dose of 4 mg/kg/day. Tonic seizures ceased after four days of administration. Because spasms and tonic seizures did not occur for two months, her parents began weaning off VPA, CLB, and TPM when she was 350 days old, ultimately discontinuing all antiepileptic drugs except CBD supplementation. She remained seizure-free until tonic seizures recurred with a high fever when she was approximately 600 days old. The CBD supplement dosage was then increased, and the seizures disappeared again when the patient was 800 days old, wherein the dosage was 400 mg/day. The occurrence of interictal epileptiform discharges was reduced in the follow-up EEG when she was 770 days old. Fig. 1

### 1.2 Case 2

This report describes the case of a 2 years and 9 months old female with convulsive seizures. She had an identical twin (Case 1) with a history of epilepsy. Born at 37 weeks and 3 days of gestation, she weighed 2548 g at birth and was the first child in a spontaneous monochorionic diamniotic twin pregnancy and spontaneous vaginal delivery. Two days after Case 1 was admitted to University Hospital B, the patient experienced brief tonic seizures in the upper limbs and was admitted to the same hospital when she was 33 days old.

During her initial admission, she weighed 3454 g. She exhibited no external deformities and had normal muscle tone, but cluster of spasms and tonic seizures lasting 20 seconds were observed. Blood counts and general biochemistry were normal, and newborn screening revealed no congenital abnormalities. An MRI scan of the head showed no abnormal findings. During this admission, PB administration began when the patient was 33 days old, followed by the addition of VPA, but the seizures persisted. ACTH therapy was initiated when she was 54 days old. Despite discontinuing PB and increasing VPA dosage, along with the addition of vitamin B6, DZP, CLB, and TPM, seizure suppression was not achieved, and the patient was transferred to a specialized facility, Hospital C.

At Hospital C, exome sequencing analysis identified a heterozygous STXBP1 variant, NM_003165: c.325+5G>A, in intron 5. VPA was changed from syrup to granules, and although she was discharged, her seizures remained uncontrollable. Consequently, she was readmitted to University Hospital B. Long-term EEG, conducted when she was 269 days old, revealed hypsarrhythmia Fig. 1.

When she was 280 days old, she was given CBD supplement 1, starting at 2 mg/kg/day and titrated up weekly by 2 mg/kg/day, reaching 600 mg/day, and the number of seizure attacks decreased, but they did not stop entirely. Her parents, on their own judgment, decided to taper off VPA, CLB, and TPM, reducing the dose to zero when she was 530 days old. The occurrence of interictal epileptiform discharges was markedly reduced in the follow-up EEG when she was 770 days old (Fig. 1). When she was 881 days old, the CBD supplement was switched to a different CBD supplement 2 (LaurelCrest Capital Inc.), administered at 600 mg/day. A considerable reduction in seizures was observed immediately, leading to complete suppression within seven days. Since then, the patient has been seizure-free for four months.

## 2 Discussion

In these cases, patients with intractable epilepsy associated with STXBP1 gene mutations achieved seizure suppression with CBD. At the initial visit, the patient was diagnosed with DEE; however, it was subsequently determined that they had developed Lennox-Gastaut syndrome.

STXBP1 is involved in the release of neurotransmitters from synaptic vesicles; therefore, when the protein content is reduced owing to haploinsufficiency, the release of neurotransmitters is inhibited, resulting in abnormal excitation of the neural network and leading to epileptic seizures. This mutation is inherited in an autosomal dominant manner, and in most cases, it occurs as a de novo mutation [8], [9], [10], [11]. This mutation, NM_003165: c.325+5G>A in intron 5, is a recurrent mutation that has been reported in at least three cases of epilepsy. One of these reports also included functional analysis, which showed that it caused abnormal splicing within the intron [12].

The mechanism by which CBD suppresses epileptic seizures remains under investigation; however, it may act through various mechanisms that are different from those of conventional antiepileptic drugs.

Several possible mechanisms exist by which CBD may have exerted an effect on DEE. First, CBD acts as a functional antagonist of the GPR55 receptor and may regulate the release of neurotransmitters [13]. This mechanism may have suppressed the excessive neural activity caused by the *STXBP1* mutation. Second, ion channel regulation may be involved. The release of neurotransmitters is ion channel-dependent, and CBD inhibits Na channels and type T calcium channels, which may reduce neuronal excitability [14], [15]. It has also been suggested that CBD directly binds to GABA receptors, thereby enhancing GABAergic-evoked current. This may partially compensate for the inhibitory neurotransmission defects caused by *STXBP1* mutations [16]. Furthermore, these mechanisms may act in combination to suppress the seizures.

In the present study, monozygotic twins achieved seizure suppression with CBD supplements containing different ingredients. The certificate of analysis (COA) of both products are presented in Table1.

**Table 1:** The certificate of analysis (COA) of both products

	Supplement (1)		Supplement (2)	
	mg/g	%	mg/g	%
CBD	993.50	99.35	704.30	70.43
THC	N.D.	–	N.D.	–
CBDA	–	–	26.71	2.67
CBC	N.D.	–	16.60	1.66
CBG	N.D.	–	3.189	0.318
CBN	N.D.	–	18.44	1.844
THCV	–	–	0.367	0.037
CBT	–	–	20.76	2.076
CBL	–	–	3.099	0.310
CBNA	–	–	0.720	0.072
CBGA	–	–	0.511	0.051
Caryophyllene	–	–	9.52	0.95
α-Bisabolol	–	–	13.99	1.40
Guaiol	–	–	2.84	0.28

N.D.: Not Detected, -: No Data, CBD: Cannabidiol, THC:Δ 9-tetrahydrocannabinol, CBDA: Cannabidiolic acid, CBC: Cannabichromene, CBG: Cannabigerol, CBN: Cannabinol, THCV: Tetrahydrocannabivarin, CBT: Cannabicitran, CBL: Cannabicyclol, CBNA: Cannabinolic acid, CBGA: Cannabigerolic acid.

CBD supplement 1 contains only purified CBD and is known as an isolate. COA showed that 99.35% of the total weight was CBD, and no other cannabinoids were detected. The product can be considered similar in composition to a CBD pharmaceutical (Epidiolex®) that is approved as a prescription drug in other countries. CBD supplement 2 contains various cannabinoids and terpenoids in addition to CBD, but the illegal ingredient THC is removed. According to the COA, CBD accounted for only 70.4% of the total weight, and ten types of cannabinoids and three types of terpenoids were detected.

Products containing all the components extracted from cannabis plants are referred to as full-spectrum products, whereas products from which only illegal components are removed are designated as broad-spectrum products. Constituents such as cannabigerol (CBG) and tetrahydrocannabivarin (THCV) in these products have demonstrated antiepileptic effects [17]. CBD, other cannabinoids, and terpenes are known to exhibit a synergistic effect termed the entourage effect [18], [19]. An analysis of the UK medical cannabis user database showed a seizure suppression rate of 31.6% with CBD isolates and 94.1% with broad-spectrum CBD products [20]. In Case 2 of this study, CBD broad-spectrum products notably reduced seizures after CBD isolated products were found to be ineffective in seizure suppression, which is consistent with the aforementioned report from the UK.

In 2020, we reported the first Japanese case of drug-resistant epileptic encephalopathy that resulted in seizure resolution after the administration of CBD supplements [21]. In 2022, a cross-sectional study of 28 Japanese cases was conducted in which CBD supplementation was used for refractory epilepsy, and 53.6% of users reported seizure reduction [6]. The majority of patients using CBD supplements for seizure control in Japan, including those in this report, which has been a part of Epidiolex® clinical trials, did not have any of the three conditions (Dravet syndrome, Lennox-Gastaut syndrome, or refractory epilepsy associated with tuberous sclerosis). This is important when considering the scope of the indications for CBD pharmaceuticals.

In addition, given that Epidolex® contains almost exclusively pure CBD, it is possible to speculate that broad-spectrum and full-spectrum products may be effective in cases where Epidiolex® is not. The reason why differences in responses were observed in genetically identical twins is unknown, but these cases are important when considering treatment options. Hence, it is important that CBD supplements can be accessed concomitantly with Epidiolex®.

## 3 Conclusions

In this study, we report two cases of monozygotic twins with developmental epileptic encephalopathy owing to *STXBP1* mutations in which long-term seizure control was achieved with CBD supplementation. One patient responded to the CBD isolate, whereas the other did not, and seizure control was attained with a broad-spectrum product containing other trace components, such as CBG and THCV. This case is crucial because it suggests that CBD may be effective in conditions beyond the three indications of the clinical trial and that alternative regimens may be effective in patients who do not respond to Epidiolex®.

Funding

Manuscript submission fee of this report was funded by the Fiscal 2024 Health and Labor Sciences Research Grant, entitled “ Regulatory oversight of cannabinoid pharmaceuticals and cannabinoid products 24CA2012 (to Y.M., N.M. and I.T.) ” in part. Part of this study was also supported by the Japanese Clinical Association of Cannabinoids.

## CRediT authorship contribution statement

**Yuji Masataka:** Writing – original draft, Project administration, Methodology, Investigation, Data curation, Conceptualization. **Naoko Miki:** Writing – review & editing, Conceptualization. **Kozo Akino:** Conceptualization. **Hitoshi Yamamoto:** Validation, Conceptualization. **Ichiro Takumi:** Supervision, Funding acquisition, Conceptualization.

## Declaration of competing interest

The authors declare that they have no known competing financial interests or personal relationships that could have appeared to influence the work reported in this paper.
